# High-Performance Integrated Self-Powered PNP Hydrogel Sensor for Wearable Human Monitoring

**DOI:** 10.3390/polym18131572

**Published:** 2026-06-24

**Authors:** Jiawei Long, Pan Niu, Hongbing Li, Yong Zhang

**Affiliations:** 1 State Key Laboratory of Advanced Glass Materials, School of Materials Science and Engineering, Wuhan University of Technology, Wuhan 430070, China; 2 Center for Smart Materials and Device Integration, Wuhan University of Technology, Wuhan 430070, China

**Keywords:** PAM, integrated, ion migration, self-powered, human sensing

## Abstract

With the rapid advancement of wearable technologies, high-performance flexible sensors have garnered significant research interest. This study presents a PAM-5 hydrogel characterized by exceptional tensile strain (425%), superior compressive modulus (325 kPa), and notable ionic conductivity (1.1 S/m), serving as a robust mechanical framework and electrical foundation for developing advanced sensors. The PNP-5 integrated hydrogel sensor fabricated from this material demonstrates an extensive sensing range (2–53 kPa), remarkable sensitivity, and rapid response time (~321 ms), with its outstanding performance attributed to the synergistic structural design. Furthermore, the sensor exhibits excellent durability, maintaining consistent voltage output (~6.5 mV) across 1000 compression cycles, confirming its long-term operational stability. Through real-time monitoring of physiological signals and biomechanical movements including finger bending, respiration, and grasping, combined with spatial pressure mapping experiments using a 5 × 5 array touchpad, the device’s potential applications in wearable sensing platforms and human–machine interface systems are effectively demonstrated. This self-powered hydrogel sensor not only advances the performance metrics of flexible electronic devices but also establishes a solid experimental basis for future development of intelligent materials in health monitoring and interactive technologies.

## 1. Introduction

With the expanding applications of wearable electronic devices and flexible sensors in health monitoring, intelligent human–machine interaction, and soft robotics, there is a growing demand for high-performance, flexible, self-powered sensing materials [1,2,3,4,5,6]. Hydrogels have emerged as promising candidates for constructing flexible electronic devices and wearable sensors owing to their high water content, inherent softness and stretchability, and superior ionic conductivity [7,8,9,10,11]. Compared with conventional rigid electronic materials, hydrogels demonstrate biomimetic properties that closely resemble biological tissues in terms of mechanical flexibility and biocompatibility, which confer distinct advantages for physiological signal monitoring, electronic skin development, wearable medical devices, and human motion detection applications [12]. The ionic conduction mechanism inherent to hydrogels facilitates highly sensitive electrical responses while enabling efficient signal transduction in flexible strain or pressure sensing modalities. However, conventional hydrogel systems still encounter challenges related to mechanical robustness, long-term conductive stability, and operational durability. Particularly under prolonged usage scenarios, repeated mechanical deformation cycles, or complex environmental conditions, these materials often display susceptibility to performance degradation, signal drift, and conductive instability-factors that critically limit their practical deployment reliability in wearable electronic device configurations [13,14,15].

Current strategies for improving the performance of hydrogels primarily involve optimizing their mechanical and electrical properties by modulating network structures, incorporating functional ions or nanocomposites, and designing multilayered or integrated architectures [16,17,18]. For example, optimizing crosslinker content effectively enhances the tensile and compressive properties of hydrogels; however, excessive crosslinking diminishes flexibility and impedes ion migration, thereby reducing conductivity [19,20]. Additionally, hydrogels derived from single materials face limitations in selectively responding to specific physiological signals, which has led to increased research focus on multilayered or integrated designs [21,22,23,24]. In recent years, integrated hydrogel sensors have demonstrated highly sensitive detection capabilities for pressure, tension, and complex physiological signals through the combination of conductive layers with ion-selective components [13,25,26]. These configurations not only broaden sensing ranges and accelerate response rates but also ensure stable output during extensive cycling, guaranteeing long-term reliability. Although the potential applications of such sensors in wearable health monitoring, human motion recognition, and intelligent human–machine interaction systems have been extensively validated, challenges persist in expanding linear response ranges, minimizing response delays, and enhancing long-term cycling stability—critical scientific issues that represent key development directions for optimizing novel hydrogel materials and system integration [17,27,28]. The design of hydrogel-based flexible sensors not only advances the field of flexible electronic materials but also establishes theoretical and experimental foundations for developing high-performance, multifunctional wearable electronic systems.

In this study, we fabricated an integrated self-powered high-performance hydrogel sensor by integrating PAM hydrogel with an ion-selective gel via a three-step photopolymerization process. Through systematic modulation of the crosslinker concentration, we achieved simultaneous optimization of mechanical robustness and electrochemical performance in the hydrogel matrix. The experimental data revealed that at an optimal MBAA concentration of 0.5%, the resulting PAM-5 hydrogel demonstrated optimal equilibrium between mechanical integrity and ionic conductivity. The corresponding PNP-5 integrated sensor exhibited a maximum output voltage of 9.9 mV, maintained a broad linear detection range (2–53 kPa), and retained stable voltage output after 1000 consecutive compression cycles, thereby confirming its exceptional durability. This sensor platform enabled accurate motion monitoring capabilities and facilitated high-resolution spatial pressure mapping when configured in an array touchpad format. Our findings offer novel perspectives for advancing flexible hydrogel applications in biomedical sensing platforms, wearable electronic devices, and human–machine interface systems. Notably, the sensor’s response time was significantly reduced due to the tailored ion transport channels within the hydrogel, allowing rapid detection of transient mechanical stimuli and making it suitable for high-speed motion tracking applications. This sensor platform enabled accurate motion monitoring capabilities and facilitated high-resolution spatial pressure mapping when configured in an array touchpad format. In addition, the integration of PAM hydrogel with ion-selective gel provides a feasible strategy for balancing mechanical stability and ion transport efficiency within a single sensing platform. Such a structural design is beneficial for maintaining continuous electrical signal output during deformation while preserving the soft and flexible characteristics required for wearable applications. More importantly, the simple photopolymerization-based fabrication process may facilitate scalable preparation and further functional integration of hydrogel sensors for practical biomedical and intelligent sensing systems. Our findings offer novel perspectives for advancing flexible hydrogel applications in biomedical sensing platforms, wearable electronic devices, and human–machine interface systems.

## 2. Experimental Section

### 2.1. Materials

Acrylamide (PAM), sodium chloride (NaCl, purity > 99.9%), N,N’-methylenebisacrylamide (MBAA), 2-acrylamido-2-methyl-1-propanesulfonic acid (AMPS), and 2-hydroxy-4′-(2-hydroxyethoxy)-2-methylphenylpropanone (Irgacure 2959) were provided by Aladin Company (Shanghai, China). Copper foil and PI tape were provided by China National Pharmaceutical Group Chemical Reagent Co., Ltd. (Shanghai, China). All chemicals were used without further purification.

### 2.2. Preparation of PAM Hydrogel Solutions with Different Crosslinking Degrees and PAM Hydrogels

First, 3 g of polyacrylamide (PAM) was dissolved in 12 g of deionized water and stirred magnetically at room temperature for 30 min until completely dissolved. Then, MBAA was added to the above solution at mass fractions (relative to PAM) of 0.1%, 0.3%, 0.5%, and 0.7%, respectively. The solution was stirred for another 30 min to ensure complete dissolution of MBAA. Subsequently, an appropriate amount of sodium chloride (NaCl) was added and stirred at room temperature for 30 min until the solution was uniform. Then, an appropriate amount of photoinitiator Irgacure 2959 was added and mixed thoroughly until the solution was homogeneous. The PAM solution containing NaCl was prepared and set aside. Following the same proportion and operation steps, a second solution was prepared by adding 3 g of PAM and 12 g of deionized water, and then adding the corresponding proportion of MBAA and photoinitiator in sequence, and stirring until uniform. The PAM solution without NaCl was prepared. The PAM solution containing NaCl was added to a silicone mold and irradiated with ultraviolet light at 365 nm for 15 min to prepare PAM hydrogels with different crosslinking degrees, which were named PAM-1, PAM-3, PAM-5, and PAM-7, respectively.

### 2.3. Preparation of Ion-Selective Gel

Acrylamide (PAM), sodium chloride (NaCl, purity > 99.9%), N,N’-methylenebisacrylamide (MBAA), 2-acrylamido-2-methyl-1-propanesulfonic acid (AMPS), and 2-hydroxy-4′-(2-hydroxyethoxy)-2-methylphenylpropanone (Irgacure 2959) were supplied by Aladin Company (Shanghai, China). Copper foil and polyimide (PI) tape were obtained from China National Pharmaceutical Group Chemical Reagent Co., Ltd. (Shanghai, China). All chemical reagents were used as received without additional purification.

### 2.4. Preparation of PN Hydrogel Sensors

The PAM solution containing NaCl was poured into a preparative mold and photopolymerized under UV irradiation (365 nm) for 10 min to form the initial hydrogel network. Subsequently, an ion-selective gel precursor was introduced and photopolymerized under identical UV conditions for approximately 4 min to establish the intermediate ion-selective layer. Finally, the PAM solution devoid of NaCl was added to the mold and photopolymerized under UV irradiation for an additional 10 min to complete the fabrication of the composite hydrogel structure. The resulting hydrogel sensors with varying crosslinking densities were designated as PNP-1, PNP-3, PNP-5, and PNP-7, respectively.

### 2.5. Characterization

Mechanical properties were tested using an electronic tensile machine, and ionic conductivity was measured using an electrochemical workstation. Copper foils (approximately 0.05 mm thick) were attached to both sides of the PNP sensors and used as electrodes for electrical property characterization. The collected energy and sensing signals were recorded using a digital multimeter (DM6500, Keithley, Solon, OH, USA).

## 3. Results and Discussion

**Figure 1:**
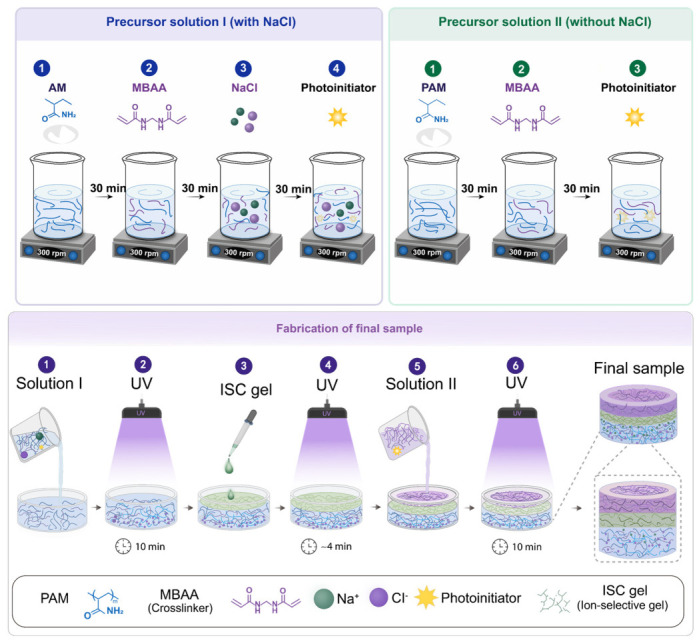
Schematic illustration of the preparation of PAM hydrogel precursor solutions and the layer-by-layer UV-induced fabrication of the integrated hydrogel sensor.

Figure 1 schematically illustrates the preparation methodology for both the PAM hydrogel solution and the PNP-integrated hydrogel sensor. The PAM hydrogel is synthesized through photopolymerization, wherein ultraviolet (UV) irradiation initiates monomer polymerization to form a crosslinked 3D network structure. This approach allows precise control over the hydrogel’s network density, pore structure, and mechanical properties, which are critical for tuning its ionic conductivity and flexibility for sensor applications. The fabrication process of the PNP-integrated hydrogel sensor adheres to a three-stage polymerization protocol: initially, a NaCl-containing PAM hydrogel solution undergoes photopolymerization; subsequently, a cation-selective gel precursor solution is deposited onto the substrate and UV-polymerized to form an ion-selective membrane layer demonstrating selective responsiveness to target ions; finally, a NaCl-free PAM hydrogel layer is overlaid and photopolymerized to complete the multilayer architecture, thereby achieving integration of mechanical robustness and functional performance within the composite hydrogel system. Such a multilayer design not only ensures effective ion transport and signal transduction across the sensor but also improves long-term stability under repeated mechanical deformation, enabling reliable performance for wearable and flexible electronic applications.

**Figure 2:**
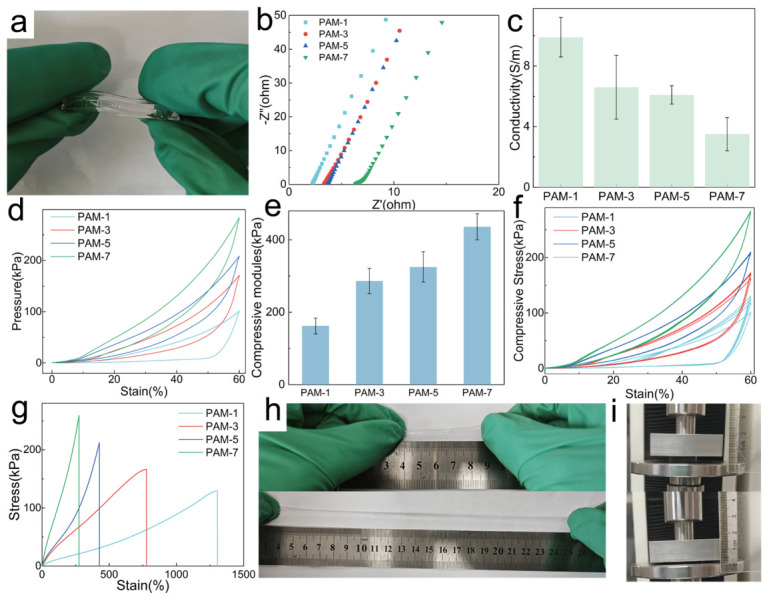
(**a**) Optical image of the integrated hydrogel. (**b**) Nyquist plots of PAM hydrogels with different crosslinking degrees. (**c**) Ionic conductivity of PAM hydrogels. (**d**) Compressive stress–strain curves. (**e**) Compressive modulus. (**f**) Cyclic compressive stress–strain curves. (**g**) Cyclic compressive stress–strain curves under extended strain. (**h**) Optical images of tensile tests. (**i**) Optical images of compressive tests.

Figure 2 illustrates the structural characteristics, ionic conductivity, and mechanical properties of polyacrylamide (PAM) hydrogels with varying crosslinking densities. Figure 2a shows an optical microscopy image of the monolithic hydrogel, indicating strong interfacial adhesion in the fabricated integrated gel structure. No obvious interfacial defects, cracks, or delamination phenomena were observed, suggesting that the sequential photopolymerization process effectively promoted interlayer bonding and contributed to the structural integrity of the integrated hydrogel system. Impedance spectroscopy analysis (Figure 2b) demonstrates that PAM hydrogel impedance increases progressively with higher crosslinking density, which suggests hindered ion transport in hydrogels with elevated crosslinking levels. The ionic conductivity can be quantitatively calculated from the impedance data using the following equation:

(1) $$\sigma \;=\;\frac{l}{R\times S}$$

where σ represents the ionic conductivity, R represents the resistance, S represents the cross-sectional area of the hydrogel, and *l* represents the thickness of the hydrogel.

The corresponding ionic conductivity (Figure 2c) demonstrates a decreasing trend. Notably, the ionic conductivity of PAM-7 decreases markedly to 1.1 S/m, which can be attributed to the densification of the network structure that obstructs ionic channels. This phenomenon indicates that excessive crosslinking restricts the mobility of hydrated ions and reduces the effective free volume available for ion migration, thereby increasing transport resistance throughout the hydrogel matrix. For mechanical characterization, tensile specimens were fabricated in dumbbell-shaped configurations with dimensions of 50 mm × 8.5 mm × 2 mm, and tensile tests were conducted at a crosshead speed of 10 mm min^−1^. Compression samples were prepared as solid blocks measuring 20 mm × 20 mm × 4 mm, with compression testing performed at 5 mm min^−1^. Optical images from the tensile test (Figure 2h) and compression test (Figure 2i) reveal that the hydrogel retains structural integrity under substantial strain, indicating superior toughness and reversible deformation capacity. The compression modulus was calculated using the following formula:

(2) $$E=\frac{\sigma }{\varepsilon }=\frac{F/A}{\Delta L/L}$$

where σ represents stress, ε represents strain, F represents the compressive force, A represents the cross-sectional area of the hydrogel perpendicular to the direction of F, ΔL is the displacement, and L is the original length in the compression direction.

The compressive stress–strain curves (Figure 2d) and corresponding compressive moduli (Figure 2e) demonstrate that increased crosslinking density in PAM hydrogels correlates with significantly enhanced rigidity, establishing a direct relationship between crosslinking density and mechanical load-bearing capacity. The denser three-dimensional network structure formed in highly crosslinked hydrogels enables more effective stress dispersion under external forces, resulting in elevated compressive modulus values. Furthermore, the increased number of crosslinking points strengthens intermolecular interactions within the polymer chains, allowing the hydrogel network to withstand higher external loads without structural collapse. Cyclic compression experiments (Figure 2f) further reveal that these highly crosslinked materials exhibit superior deformation recovery capabilities and stable mechanical responses across multiple compression cycles, which suggests that the robust network architecture provides an intrinsic restoring force mechanism while mitigating material fatigue effects. Concurrently, experimental data show progressive reduction in fracture elongation with increasing crosslinking density (Figure 2g), indicating diminished flexibility in highly crosslinked states. These findings confirm that precise control over crosslinking density allows targeted modulation of mechanical properties while maintaining ionic conductivity. High crosslinking densities enhance mechanical robustness and cyclic stability, whereas lower densities favor extensibility and flexibility [29]. Therefore, an optimal crosslinking density is essential for balancing mechanical strength and ionic transport efficiency, which is a critical requirement for achieving high-performance flexible sensing systems. This mechanistic understanding offers a viable material design strategy and theoretical framework for developing hydrogel-based applications in flexible electronic devices, wearable sensors, and soft robotics.

**Figure 3:**
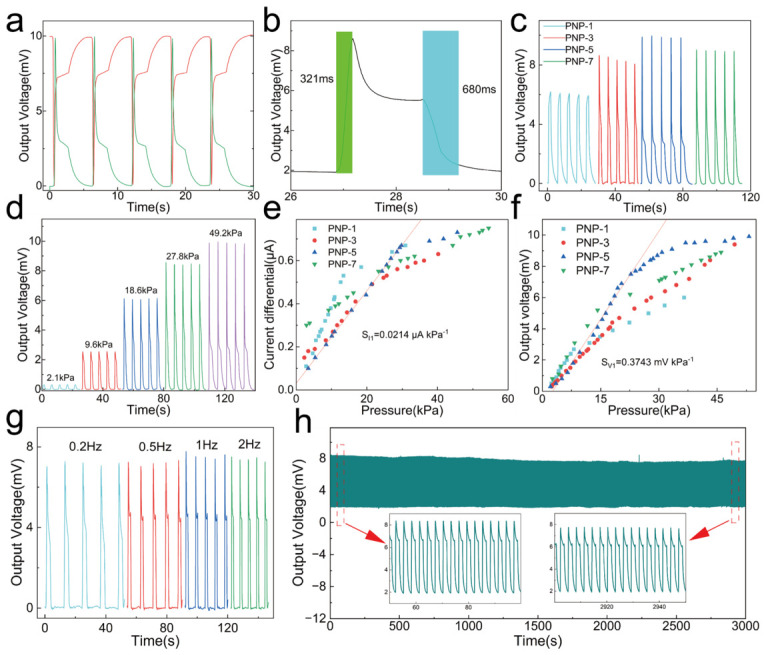
(**a**) Output voltage of PNP-5 hydrogel under forward and reverse external circuits. (**b**) Response and recovery times of PNP hydrogels. (**c**) Maximum output voltage of PNP hydrogels with different crosslinker contents. (**d**) Output voltage of PNP-5 hydrogel under varying applied pressures. (**e**) Current versus pressure relationship for PNP hydrogels with different crosslinker contents. (**f**) Voltage versus pressure relationship for PNP hydrogels with different crosslinker contents. (**g**) Frequency-dependent stability of the output voltage of PNP-5 hydrogel. (**h**) Durability test of PNP-5 hydrogel under 1000 cycles.

An integrated hydrogel was fabricated via a three-step photopolymerization approach, and its sensing performance was systematically evaluated. Figure 3 presents the electrical output characteristics and dynamic response of PNP hydrogels with varying crosslinker concentrations. When mechanical pressure is applied to the PNP hydrogel, cations and anions within the polyacrylamide (PAM) layers on both sides translocate toward the opposing interface. A schematic illustration of ion migration and voltage generation is shown in Figure S1. In the absence of external pressure, the ions within the PNP hydrogel are relatively uniformly distributed, and the system remains in an electrically neutral equilibrium state, resulting in a near-zero output voltage. When external pressure is applied, the hydrogel network undergoes compressive deformation, which promotes ion redistribution. Since the intermediate ion-selective layer allows only cations to pass through while restricting the transport of anions, an ion concentration gradient is established across the device, leading to the generation of a measurable potential difference. Upon release of the external pressure, the hydrogel network gradually recovers its original structure, and the ions tend to redistribute uniformly again. Consequently, the charge separation effect weakens, and the output voltage gradually returns to its initial level. Upon reaching the central ion-selective gel layer, these ions establish a potential difference across the hydrogel owing to its preferential cation permeability and anion exclusion. Following force release, ion migration reverses direction governed by concentration gradients and the established potential difference, thereby restoring electrostatic equilibrium and enabling self-powered operation [30]. As shown in Figure 3a, the PNP-5 hydrogel generates a stable output voltage under both forward and reverse circuit configurations. The temporal analysis in Figure 3b reveals response (≈321 ms) and recovery times (≈680 ms), which are attributed to the structural integration facilitating rapid electrical signal transduction and relaxation dynamics.

The effect of crosslinker content on the output performance of the hydrogel is shown in Figure 3c. As the crosslinker content increases, the maximum output voltage gradually rises, with the PNP-5 hydrogel exhibiting the highest peak voltage (9.9 mV). However, as the crosslinking density increases further, the maximum output voltage decreases. This occurs because at low crosslinking densities, the hydrogel’s mechanical properties are insufficient, causing the voltage to reach saturation under relatively small forces. These results demonstrate that crosslinking density plays a significant role in regulating the sensor’s output signal [31,32]. The corresponding current-pressure relationship (Figure 3e) and voltage-pressure relationship (Figure 3f) further quantify the pressure sensitivity of the hydrogel, with sensitivities of S_I_ = 0.0214 μA kPa^−1^ and S_V_ = 0.021 mV kPa^−1^. This indicates its potential for application in micro-pressure sensing. Figure 3d shows that the output voltage of the PNP-5 hydrogel increases stepwise with rising applied pressure. Upon reaching a critical threshold, the rate of voltage increase slows down and eventually plateaus, indicating that ion migration has reached saturation. In the low-pressure region, the applied pressure effectively compresses the hydrogel network, promoting ion redistribution and directional ion migration across the ion-selective layer. As a result, the output voltage increases rapidly with increasing pressure. As the pressure continues to increase, the hydrogel network becomes progressively compacted, reducing the free volume and deformable space within the system. Consequently, the pressure-induced changes in ion transport pathways become less significant, leading to a reduced degree of ion redistribution. Meanwhile, the ion concentration gradient generated by pressure gradually approaches a steady state. Therefore, the rate of voltage increase decreases, and the output signal exhibits a saturation tendency. Previous studies have also shown that, at higher pressure levels, the further deformation capability of the sensing structure becomes limited or the effective contact area approaches saturation, resulting in a lower signal variation rate and reduced sensitivity [33]. Considering its overall response range and electrical output signal, the PNP-5 hydrogel demonstrates optimal comprehensive performance, and thus it was selected for subsequent testing. Frequency-dependent tests (Figure 3g) show that it can produce stable voltage outputs within the frequency range of 0.2–2 Hz, indicating reliable response characteristics under different dynamic loading conditions. Durability tests (Figure 3h) further reveal that the output voltage remains essentially stable after 1000 compression cycles, with an output voltage of 6.5 mV and no significant attenuation observed. This demonstrates the material’s excellent mechanical-electrical stability and long-term reliability. Furthermore, the integration of the ion-selective layer and optimized crosslinker concentration allows fine-tuning of both sensitivity and dynamic response, ensuring that the sensor maintains high performance under various pressure magnitudes and loading rates. Such design flexibility makes the PNP-5 hydrogel a promising candidate for wearable pressure monitoring, interactive robotics, and multi-modal tactile sensing applications. The systematic evaluation highlights the correlation between network structure, ion migration dynamics, and electrical output, providing a blueprint for engineering next-generation self-powered hydrogel sensors with tailored electromechanical properties. By tailoring the hydrogel network structure through different crosslinker contents and designing it into an integrated structure, the output voltage, pressure sensitivity, and response speed can be effectively optimized while maintaining good frequency adaptability and cyclic stability. The sensor exhibits outstanding mechanical properties, meeting the requirements for practical applications. Table S1 compares its performance with recently reported representative self-powered hydrogel pressure sensors. The results show that the PNP-5 sensor combines a wide pressure detection range (2–53 kPa), high sensitivity (0.374 mV kPa^−1^), fast response/recovery speed (0.32/0.68 s), and excellent cycling stability (1000 cycles). These findings indicate that the constructed integrated architecture effectively balances sensing performance and long-term stability.

**Figure 4:**
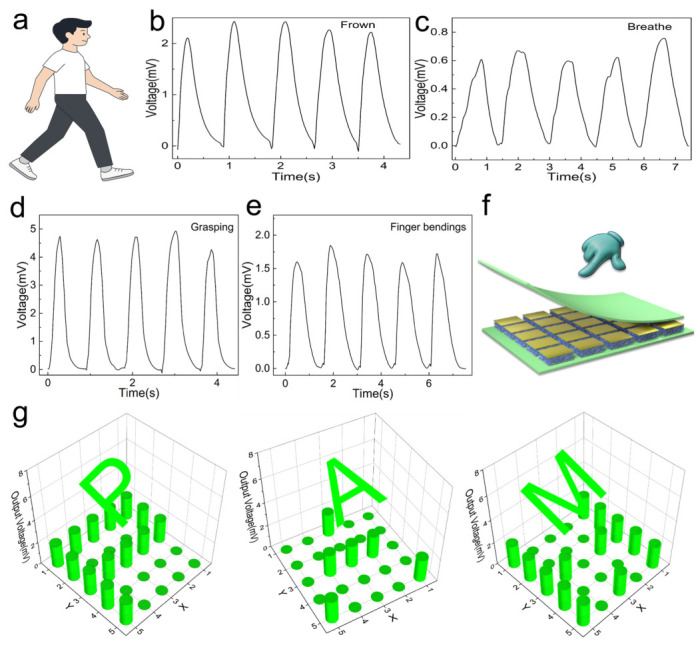
(**a**) Human motion monitoring. (**b**–**e**) Electrical responses of PNP-5 hydrogel during frowning, breathing, grasping, and finger bending activities. (**f**) Schematic illustration of a 5 × 5 ionic hydrogel touchpad based on PNP-5. (**g**) Electrical responses of the ionic touchpad under finger sliding.

In addition to tactile perception functionality, this device can also monitor multiple human physiological signals (Figure 4a), including frowning, respiration, grasping movements, and finger bending, as demonstrated in Figure 4b–e. Experimental results indicate that the sensor successfully captures distinct electrical signal characteristics for each type of physiological activity, demonstrating its potential for dynamic physiological monitoring. To further validate the sensor’s capabilities in spatial resolution and multipoint detection, we designed a 5 × 5 array touchpad where each unit consists of PNP-5 hydrogel with dimensions of 1 cm × 1 cm. To prevent moisture evaporation from the hydrogel and ensure long-term stability, the entire array was encapsulated using PDMS. By writing the letters “P”, “A”, and “M” on the touchpad with a finger, the sensor array accurately generated corresponding signal histograms (Figure 4g), where changes in output voltage corresponded to the positions of applied external forces on each unit. These results not only verify the effectiveness of the sensor in high-resolution tactile perception but also demonstrate its application potential in wearable health monitoring scenarios, including motion recognition, posture monitoring, and physiological status tracking. Furthermore, the sensor array exhibited excellent signal distinguishability and spatial recognition capability, enabling precise localization of external stimuli. The stable and repeatable electrical responses obtained during different motion states further highlight its suitability for real-time human–machine interaction and intelligent sensing applications. Additionally, the rapid signal response and reliable spatial mapping performance indicate that the sensor can effectively translate complex mechanical stimuli into distinguishable electrical outputs, supporting advanced sensing and interactive functionalities. This study provides robust experimental evidence and design insights for applying flexible hydrogel sensors in intelligent health monitoring devices.

## 4. Conclusions

The PAM-5 hydrogel demonstrates exceptional mechanical properties including a high tensile strain (425%), excellent compressive modulus (325 kPa), and superior ionic conductivity (1.1 S/m), establishing a robust electromechanical foundation for developing high-performance flexible sensors. The optimized balance between structural stability and ion transport capability enables the hydrogel to maintain reliable performance under various mechanical deformation conditions, highlighting its suitability for practical wearable applications. These synergistic properties result from the optimized hydrogel network architecture, which effectively balances mechanical strength, flexibility, and ion transport efficiency. The PNP-5 integrated hydrogel sensor fabricated from this material exhibits a wide sensing range (2–53 kPa), remarkable sensitivity, and fast response time (~321 ms), which can be attributed to its judiciously engineered structural design. Notably, the sensor maintains stable voltage output (up to ~6.5 mV) after enduring 1000 cyclic compressions, thereby confirming its long-term operational stability. Real-time monitoring capabilities for human physiological signals and motions (including finger bending, respiration, and gripping), combined with spatial pressure recognition experiments using a 5 × 5 touchpad array, further validate its potential applications in wearable sensing platforms and human–machine interface systems. Moreover, the hydrogel’s combination of mechanical resilience, fast ionic transport, and self-powered operation offers a versatile platform for integrating additional sensing modalities, such as temperature or chemical detection, enabling multifunctional wearable devices. These features not only expand the scope of real-time physiological monitoring but also provide design guidelines for scalable production of smart, adaptive electronic skins. Furthermore, the simple fabrication strategy and excellent reproducibility of the proposed system provide opportunities for large-scale manufacturing and practical deployment in next-generation flexible electronics. This self-powered hydrogel sensor configuration not only advances the performance metrics of flexible electronic devices but also provides an experimental framework for developing next-generation functional materials tailored for smart health monitoring and interactive technologies.

## Data Availability

The data supporting the plots in this study and other findings of this study are available from the corresponding author upon reasonable request.

## Supplementary Materials

The following supporting information can be downloaded at: https://www.mdpi.com/article/10.3390/polym18131572/s1, Figure S1: Schematic illustration of the sensing mechanism; Table S1: Comparison of this work with previously reported pressure sensors. References [30,34,35,36,37,38,39] are cited in the Supplementary Material.
