# Supplementary material for: High-Performance Integrated Self-Powered PNP Hydrogel Sensor for Wearable Human Monitoring
Source: Polymers (Basel). 2026 Jun 24;18(13):1572. doi: 10.3390/polym18131572 (PMC13364268; doi:10.3390/polym18131572)
Supplement: Supplementary file 1 [file polymers-18-01572-s001.zip › polymers-4349240-supplementary.pdf]

High-Performance Integrated Self-Powered PNP Hydrogel Sensor for Wearable Human Monitoring

Jiawei Long [1,2], Pan Niu [1,2], Hongbing Li [1,2] and Yong Zhang [1,2,*]

[1] State Key Laboratory of Advanced Glass Materials, School of Materials Science and Engineering, Wuhan University of Technology, Wuhan 430070, China
[2] Center for Smart Materials and Device Integration, Wuhan University of Technology, Wuhan 430070, China

* Correspondence: zhangyong123@whut.edu.cn

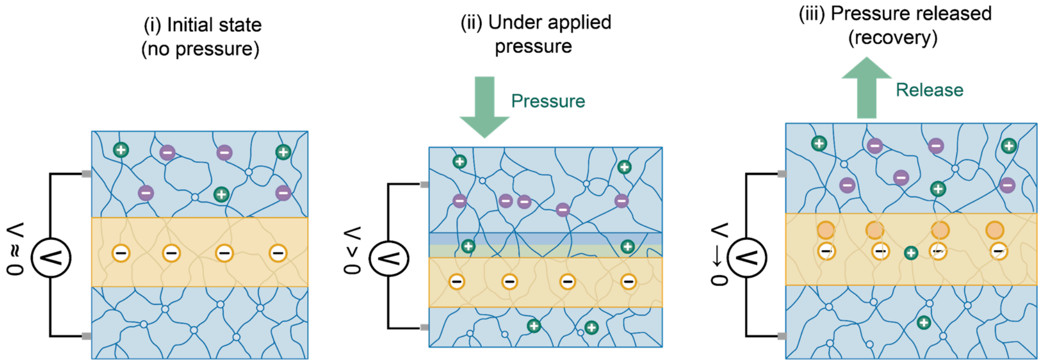

Figure S1 Schematic illustration of the sensing mechanism

Table S1 Comparison of this work with previously reported pressure sensors

Materials	Response range(kPa)	Sensitivity	Respond time /recover time	Stability
MXene/PVA[1]	0-50	0.095mV/kPa	0.47/0.53 s	1000
Ag@PDA/（CSMA-PAM）[2]	0-2.15	0.07 kPa^{-1}	0.13/0.13 s	500
STENG[3]	0-101.2	0.013 kPa^{-1}	/	20000
PKAZ[4]	1-50	0.35 kPa^{-1}	0.2/0.2 s	100
PDMAEMA/PNIPAM[5]	0-2500	14 mV/MPa	1.2/1.21 s	200
OEGA:MA[6]	0-12	0.074	0.36/0.27 s	500
PDA-PVA/PEG[7]	0-30	0.57 kPa^{-1}	.0.21/0.33 s	2000
This work	0-55	0.374mV/kPa	0.32/0.68 s	1000

Addition to the References:

1. Zhang, H.; Fang, Y.; Chen, W.; Zhang, Y. Controlled Ion Immigration in MXene-PVA Composites for Self-Powered Pressure Sensor. Chem. Eng. J. 2025, 508, 161039.
2. Wu, G.; Shi, W.; Liu, M.; Liang, L.; Wang, T.; Zhang, J.; Chen, J.; Liang, Y.; Tang, W.; Li, H. Multifunctional Strain/Pressure Sensor Based on Ag@polydopamine Nanohybrid Methacrylamide Chitosan/Polyacrylamide Hydrogel for Healthcare Monitoring. ACS Appl. Mater. Interfaces 2025, 17, 9879–9890.
3. Pu, X.; Liu, M.; Chen, X.; Sun, J.; Du, C.; Zhang, Y.; Zhai, J.; Hu, W.; Wang, Z.L. Ultrastretchable, Transparent Triboelectric Nanogenerator as Electronic Skin for Biomechanical Energy Harvesting and Tactile Sensing. Sci. Adv. 2017, 3, e1700015.
4. Song, X.; Guo, J.; Zhang, Y.; Guan, F.; Tao, J.; Yao, Q.; Ji, X. Multi-Network Hydrogel-Based Stretchable, Self-Adhesive, and Self-Healing Self-Powered Flexible Sensors for Multi-Scale, Dynamic and Static Strain/Pressure Sensing. Colloids Surf., A 2025, 708, 136035.
5. Sun, D.; Peng, C.; Tang, Y.; Qi, P.; Fan, W.; Xu, Q.; Sui, K. Self-Powered Gradient Hydrogel Sensor with the Temperature-Triggered Reversible Adhension. Polymers 2022.
6. Jiang, N.; Chen, G.; Zhou, F.; Ma, B.; Zhao, C.; Liu, H. A Dual-Mode Wearable Sensor with Electrophysiological and Pressure Sensing for Cuffless Blood Pressure Monitoring. J. Mater. Chem. C 2024, 12, 15915–15923.
7. Xin, Q.; Hu, D.; Zhou, L.; Yang, G.; Liang, S.; Lin, J. Polydopamine-Based Multifunctional Hydrogel for Flexible Supercapacitor and Pressure Sensor Applications. ACS Appl. Electron. Mater. 2023, 5, 3756–3764.
